# Long-Term Outcomes of Endovascular Aortic Repair with Parallel Chimney or Periscope Stent Grafts for Ruptured Complex Abdominal Aortic Aneurysms

**DOI:** 10.3390/jcm14010234

**Published:** 2025-01-03

**Authors:** Reinhard Kopp, Lukas Stachowski, Gilbert Puippe, Alexander Zimmermann, Anna-Leonie Menges

**Affiliations:** 1 Department of Vascular Surgery, University Hospital Zurich, 8091 Zurich, Switzerland; lukas.stachowski@usz.ch (L.S.); alexander.zimmermann@usz.ch (A.Z.); anna-leonie.menges@usz.ch (A.-L.M.); 2 Section of Vascular and Endovascular Surgery, University Hospital Regensburg, 93053 Regensburg, Germany; 3 Department of Diagnostic and Interventional Radiology, University Hospital Zurich, 8091 Zurich, Switzerland; gilbert.puippe@usz.ch

**Keywords:** ruptured abdominal aortic aneurysm, endovascular aortic repair, parallel grafts, chimney, periscope, gutter endoleak, parallel stent graft, EVAR

## Abstract

**Background**: The parallel stent graft endovascular aortic repair (PGEVAR) technique is an off-the-shelf option used for elective complex abdominal aortic aneurysm repair with acceptable outcome results, as reported so far. The PGEVAR technique, using chimney or periscope parallel grafts, can also be used for patients with ruptured complex abdominal aortic aneurysms. However, only few data about the mid- to long-term outcomes are available. **Methods**: Data from patients treated between August 2009 and July 2023 with the PGEVAR technique for ruptured complex abdominal aortic aneurysms were analyzed. The endpoints of this study were primary and secondary technical success, perioperative mortality, rate of proximal type 1a (gutter) endoleaks (T1aEL), and overall and aneurysm-related survival. Secondary endpoints were major adverse events, durability of parallel grafts, and factors associated with overall survival. **Results**: Twenty patients (mean age: 77 ± 9 y; 18 male) with ruptured complex abdominal aortic aneurysm were treated, receiving PGEVAR for ruptured juxtarenal (n = 11), suprarenal (n = 7), or distal thoracoabdominal Crawford IV aortic aneurysms (n = 2) with a mean diameter of 82 ± 18 mm (range 59–120). The patients had PGEVAR with implantation of 39 parallel grafts (1.95 PGs per patient; 23 chimney and 16 periscope) for revascularization of the celiac artery (n = 3), superior mesenteric artery (n = 9), and renal arteries (n = 27). Three patients had delayed PG implantation within 10 days. Primary technical success was 15/20 (75%) with five patients having an early proximal T1aEL, three of them having successful reintervention (secondary success rate: 18/20; 90%), with no persistent bleeding. Two patients had late T1aELs. The presence of an early T1aEL was related to the number of PGs (≥2) implanted (*p* = 0.038) or insufficient aortic SG oversizing (*p* = 0.038). In-hospital mortality was 1/20 (5%). Perioperative mortality up to 32 days was 3/20 (15%), with two further late aneurysm-related deaths and eight late aneurysm-unrelated deaths (overall mortality 13/20; 65%) during follow-up (median 34 months; range 1–115). Major adverse events were observed in 11 (55%) patients. Secondary parallel stent graft patency at 1 and 3 years was 97.4 and 94.1%. During follow-up, aneurysm sac behavior was determined in 19 patients, which showed diameter progression (n = 3), stable aneurysm disease (n = 3), and aneurysm diameter regression in 13 (68.4%) patients. Overall survival was 75% after 1 year, and 53% and 22% after 3 and 5 years. Factors associated with overall long-term survival were age < 80 years (*p* = 0.037), juxtarenal aneurysms (*p* = 0.023), the absence of major adverse events (*p* = 0.025), and aneurysm sac regression (*p* = 0.003). **Conclusions**: Treatment of ruptured complex abdominal aortic aneurysm with the PGEVAR technique is associated with acceptable perioperative and long-term outcomes with high PG patency rates. Early proximal T1aELs are observed with a relevant frequency, requiring early reintervention with successful sealing of most relevant endoleaks. To note, limitation of the number of parallel stent grafts implanted at the proximal aortic sealing sites, sufficient PG sealing length, and adequate main aortic SG oversizing are most relevant to avoid T1a (gutter) ELs. The selection of juxtarenal aortic aneurysms and evidence for aneurysm sac diameter regression after PGEVAR had a prognostic impact.

## 1. Introduction

In recent years, several innovative improvements have been made for aortic aneurysm treatment, particularly in the field of endovascular aneurysm repair (EVAR) techniques [1,2]. These assumed less-invasive EVAR techniques seem to offer new therapeutic options for several patients, with severe comorbidities considered unfit for open surgical repair [3,4,5]. While fenestrated and branched EVAR (F/BEVAR) usually represent the first choice for suitable elective cases with complex aortic aneurysms, the management of ruptured aortic aneurysms, especially those lacking an adequate proximal sealing zone, remains as a substantial challenge [6,7].

Initially introduced by Greenberg RK et al. (2003) and adapted by Criado FJ (2007), parallel graft EVAR (PGEVAR) offers an adaptable off-the-shelf option for the treatment of complex aortic aneurysms [8,9,10,11]. The initial techniques, chimney or snorkel, were added by periscope and the sandwich technique, thus providing a wide range of technical treatment options for complex aortic morphologies [12,13,14,15]. The F/BEVAR treatment has limitations due to anatomical constraints and manufacturing delays but PGEVAR can provide a potential alternative, particularly in urgent or emergency settings [16,17]. However, long-term device-related complications, such as proximal type 1a endoleaks (T1aELs)—so-called gutter endoleaks with proximal seal failure—remain a major concern, especially for ruptured aortic aneurysms [18,19].

The literature lacks comprehensive long-term outcome data for patients undergoing PGEVAR following aortic aneurysm rupture. Consequently, this paper aims to fill this gap by presenting a retrospective analysis investigating the durability of PGEVAR and the long-term outcomes of patients treated for ruptured juxtarenal, suprarenal, and distal thoracoabdominal aortic aneurysms.

## 2. Patients and Methods

### 2.1. Patients

For this retrospective data analysis, all patients who presented with a ruptured complex aortic aneurysm between August 2009 and July 2023 were screened for eligibility, with the inclusion of patients who underwent PGEVAR treatment for juxtarenal, suprarenal, and distal thoracoabdominal Crawford IV aortic aneurysms. Rupture was defined as the presence of hematoma outside the aortic wall in close relation to the defined aortic aneurysm in the primary CTA scan.

Juxtarenal abdominal aortic aneurysms have a short ≤4 mm or no infrarenal aortic neck; suprarenal aortic aneurysms involve both renal arteries just below the superior mesenteric artery (SMA); and distal thoracoabdominal aortic Crawford IV aneurysms include all of the renovisceral arteries with an aortic aneurysm extension below the diaphragm, not involving the thoracic aorta. Patients were included if they were treated with the PGEVAR technique during the first or second step, with the second step performed during the same hospital stay and within 2 weeks after the primary aortic intervention.

Excluded were non-ruptured symptomatic aneurysms, ruptured thoracoabdominal aortic aneurysms classified as Crawford I–III and V aneurysms, mycotic aortic aneurysms, ruptured aneurysms with aortic fistula, ruptured aortic dissections, localized traumatic aortic dissections, ruptured aneurysms caused by prosthetic graft/stent graft infections, or patients with aneurysms caused by genetic disorders. Also, patients having open, hybrid aortic repair or endovascular repair without the use of parallel grafts or those who underwent palliation were excluded, as well as patients who withdrew or declined their consent.

Preexisting comorbidities were taken from the patient diagnostic clinical documents, including hypertension requiring medication, coronary artery disease (prior myocardial infarction, history of coronary bypass, or stenting), chronic respiratory disease requiring medication, renal insufficiency (estimated glomerular filtration rate, eGFR below 30 mL/min/1.73 m^2^), prior stroke, and history of prior aortic repair.

### 2.2. Study Outcome Criteria

The primary outcome criteria were primary technical success (defined as a complete exclusion of ruptured aortic aneurysms from perfusion without persistent bleeding and without T1EL or T3EL), perioperative, overall, and aneurysm-related mortality, and overall survival. Secondary technical success was evaluated after early reintervention for high-flow T1aELs. Secondary outcome criteria were major adverse events, parallel graft patency, access site complications requiring reintervention, aneurysm diameter behavior, and factors influencing overall survival.

### 2.3. Endovascular Aortic Repair and Parallel Stent Graft Technique

The parallel stent graft technique included single or multiple (up to 4) renovisceral stent graft implantations in a chimney or periscope configuration. The chimney or periscope parallel stent grafts were then fixed and sealed by the proximal or distal end of the main aortic stent graft, an additional cuff, or in-between the proximal aortic stent graft and an additionally overlapping stent graft (the sandwich technique). Most parallel stent grafts were then realigned using additional self-expandable stents to improve visible angulations and to prevent parallel stent graft stenosis or non-alignment.

**Figure 1:**
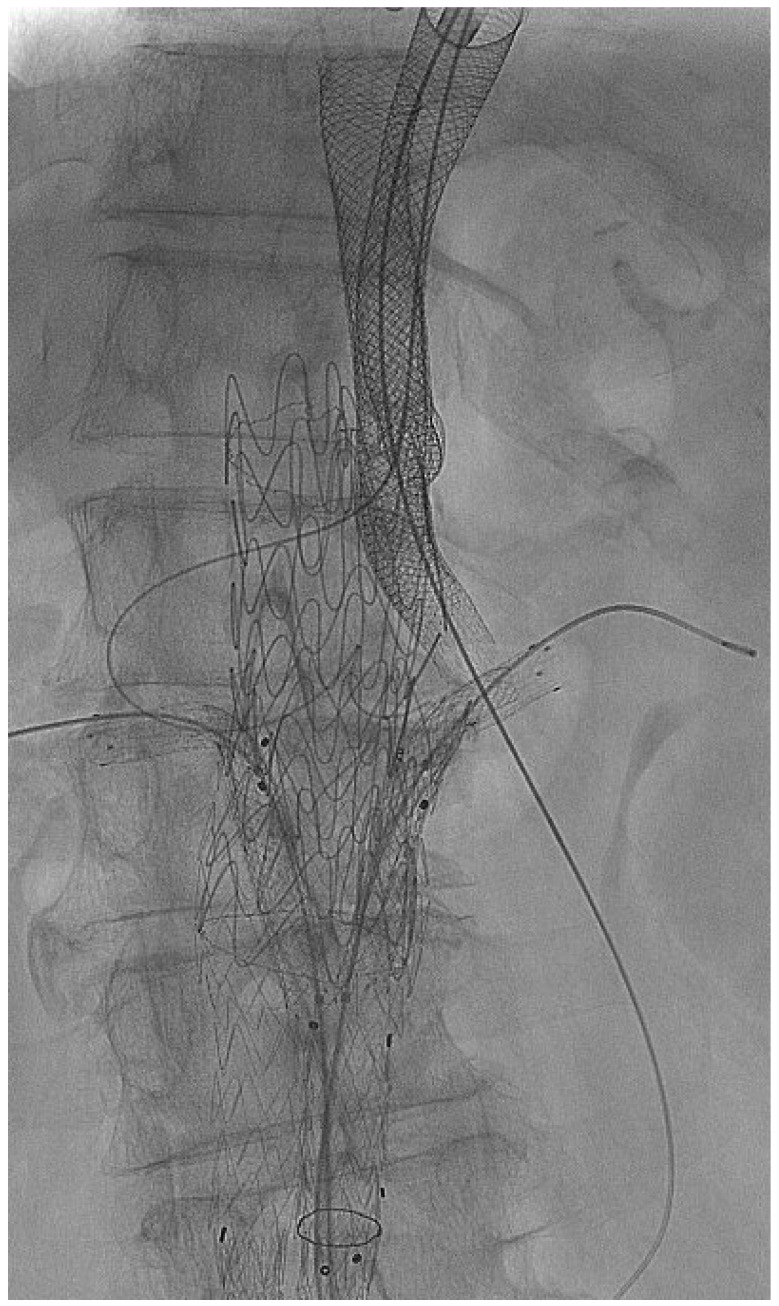
The PGEVAR technique for treatment of a ruptured suprarenal abdominal aortic aneurysm is presented. Two downward oriented chimney parallel stent grafts are implanted into the celiac artery and the SMA and two upward directed periscope parallel stent grafts for perfusion of renal arteries, sealed by an additional tube aortic stent graft.

According to our experience, the publications of several studies and recent ESVS guidelines recommend that no more than two parallel stent grafts should be positioned in the proximal or distal aortic landing zones to prevent incomplete sealing and proximal or distal T1a (gutter) or T1b endoleaks. The implantations of 3 or 4 renovisceral parallel stent grafts were performed by separate proximal and distal implantation of parallel stent grafts, with no more than 2 stent grafts at every sealing site (Figure 1). Oversizing of the most proximal sealing aortic stent grafts by at least 30% and an aortic sealing length of 20 mm was intended in a non-severely angulated (<60 degrees) visceral aortic segment. Parallel stent graft diameters were adjusted to the diameter of the target vessels with 10–15% oversizing.

### 2.4. Data Acquisition

Patients were identified in the institutional clinical information system (KISIM 5.1.0.3; CISTEC AG, Zurich, Switzerland) and the patient’s baseline characteristics and data related to aortic aneurysm characteristics with aortic, aortoiliac, and renovisceral vessel measurements were documented. Peri- and postoperative outcomes were evaluated by assessing overall mortality in hospital-, perioperative-, and aneurysm-related mortality, days in the ICU, length of hospital stay, and postoperative complications. During follow-up, patients were seen in our outpatient department or, if required, the latest data were obtained from external hospitals or by contacting the patients or their relatives. Data acquisition was in accordance with the Declaration of Helsinki.

### 2.5. Pre- and Postoperative CTA Imaging and Evaluation Processes

Pre- and postoperative CTAs were analyzed using a 3-dimensional workstation (XERO Viewer 8.1.2, Agfa HealthCare N.V., Mortsel, Belgium). Description of proximal neck quality was performed by measuring the length of proximal and distal landing zones, diameter, thrombus formation, and longitudinal aortic angulation based on CTA imaging. Endoleak classification and morphological aortic analysis were independently performed by the first and senior authors, and inconsistencies were discussed before reaching an agreement.

### 2.6. Definitions

Definitions of juxtarenal, suprarenal, and distal thoracoabdominal aortic aneurysms corresponding to Crawford IV aneurysms were already mentioned. Endovascular aortic repair and endoleaks were characterized according to the reporting standard for endovascular aortic therapy involving renovisceral arteries, including the description of type 1–4 endoleaks. Type 1a or 1b endoleaks were considered when antegrade (T1aEL) or retrograde (T1bEL) perfusions along and between the aortic stent graft and the parallel stent grafts were visible with perfusion of the aneurysm sac. Visible T1aELs (gutter endoleaks) based on CTA imaging were classified as early arterial high-flow endoleaks or late low-flow endoleaks during the late so-called venous imaging phase [20,21,22]. Contrast medium collections between parallel grafts and aortic stent grafts without perfusion of the aneurysm sac were not considered as a T1aEL.

Aneurysm sac behavior was classified according to changes in maximal aortic diameter between the preoperative CTA and the latest CTA during follow-up, measured perpendicular to centerline reconstructions and classified as progression with >5 mm diameter increase, stable aortic disease with −5 to + 5 mm diameter differences, or aneurysm regression with a decreased aneurysm diameter of <−5 mm.

Major adverse events were defined as a composite of perioperative death; myocardial infarction; respiratory failure requiring prolonged, ≥5 d, mechanical ventilation or reintubation; renal function decline resulting in a >50% reduction in baseline eGFR or new-onset dialysis; bowel ischemia requiring surgical resection; major stroke; or paraplegia. Perioperative and overall mortality rates and the need for reinterventions were monitored throughout the initial 32 postoperative days and until the latest follow-up. The 32 d perioperative mortality is reported because two patients died 31 and 32 days after discharge, which was unrelated to their aneurysm disease, and they had refused further treatment.

### 2.7. Follow-Up Protocol

During follow-up after PGEVAR for ruptured complex aortic aneurysm, CTA imaging was performed immediately after the intervention, prior to discharge, and after 3, 6, and 12 months, followed by annual intervals thereafter. In patients with aneurysm sac regression, abdominal imaging was alternatively performed by either CTA or contrast-enhanced ultrasound to determine aneurysm sac behavior and renovisceral perfusion at yearly intervals. For this retrospective observational cohort study, follow-up information was included up to the pre-specified study end date of 30 September 2023.

### 2.8. Details About Implanted Stent Graft Devices

During PGEVAR for ruptured complex abdominal aortic aneurysms, overall, 16 bifurcated stent grafts and 4 tube stent grafts were implanted. The aortic stent graft devices employed were Endurant (n = 12; Medtronic Inc., Minneapolis, MN, USA); cTAG (n = 3) and Aortic Excluder (n = 2; W.L. Gore & Associates, Inc., Flagstaff, AZ, USA); Zenith alpha (n = 2; Cook, William Cook Europe, Bjaeverskov, Denmark); and Lombard Minos (n = 1; Shanghai MicroPort Endovascular MedTech, Shanghai, China). Ten patients had additional implantations of proximal aortic cuffs (Endurant or Excluder aortic extensions) for short infrarenal aortic neck (so-called hostile neck) requiring proximal extension of the aortic sealing zone. For parallel graft implantation into renovisceral target arteries, self-expandable stent grafts were used (36/39 PGs; 92.3%; Viabahn, W.L. Gore & Associates, Inc., Flagstaff, AZ, USA) with frequent additional endolining using bare metal stents in 22 target vessels (wall stent, Boston Scientific AG, Solothurn, Switzerland). Most recently, balloon-expandable parallel stent grafts were used for implantations into 3 renal arteries in 3 patients (Advanta: Atrium, Merrimack, NH, USA; iCover: iVascular, Life Vascular Devices Biotech S.L., Barcelona, Spain; BeGraft, Bentley, Medical Equipment Export LLC, Seneca, SC, USA).

### 2.9. Statistical Analysis

Categorical variables are presented as counts and percentages and compared using the Chi-squared test. Continuous variables are summarized by means and standard deviation if normally distributed or by the median and interquartile range and compared using *t*-tests or the Mann–Whitney U test, respectively. Survival analysis was performed by the Kaplan–Meier method using the log-rank test to calculate statistical differences. Statistical analyses were performed using SPSS software (version 26, IBM Corp., Armonk, NY, USA) with an alpha level of 5%.

## 3. Results

### 3.1. Patients and Procedural Details

**Figure 2:**
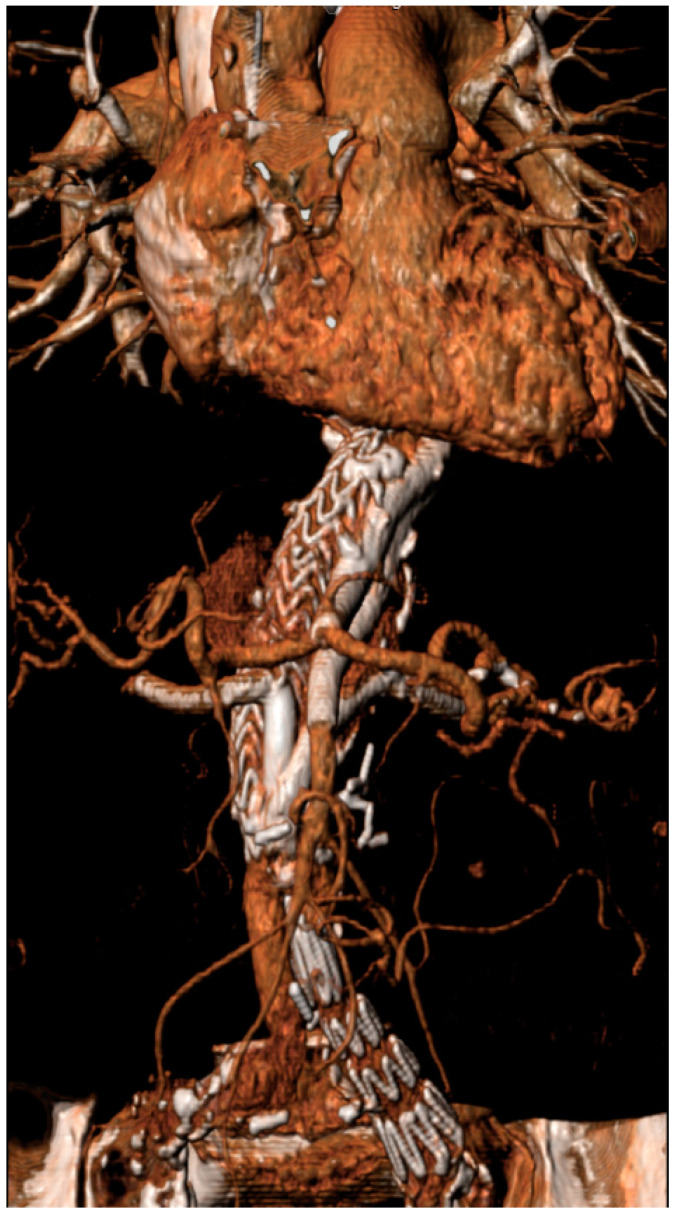
Three-dimensional reconstruction of a ruptured distal Crawford IV thoracoabdominal aortic aneurysm treated with the PGEVAR technique using 2 proximal chimney PGs to the visceral arteries (celiac artery and superior mesenteric artery) and 2 periscope PGs to the right and left renal artery, with no evidence for a proximal T1aEL or distal T1bEL.

Twenty patients (mean age: 77 ± 9 y; 18 male) with ruptured complex abdominal aortic aneurysms—juxtarenal (n = 11), suprarenal (n = 7), and distal thoracoabdominal Crawford IV aneurysms (n = 2)—with a mean diameter of 82 ± 18 mm (range 59–120) were treated using PGEVAR between August 2009 and July 2023 (Figure 2).

Nine patients had prior open aortic surgery (n = 3) or bifurcated endovascular aortic repair (EVAR; n = 6); four of them had an abdominal aortic aneurysm rupture for T1aELs after prior EVAR. Four patients presented with shock, hypotension, and anemia at hospital admission (Table 1).

Patients were treated by PGEVAR with implantations of 39 parallel stent grafts (1.95 PGs per patient; 23 chimney and 16 periscope) for revascularization of the celiac artery (CA, n = 3), SMA (n = 9), and right and left renal arteries (n = 27). Two patients required immediate intraoperative extension of the proximal sealing zone by parallel graft extension with proximal cuff implantation and complete sealing. Three patients had delayed PG implantation after EVAR for ruptured aneurysms with short infrarenal neck or suspected proximal T1aELs after 1, 3, and 10 days.

Implantations of PGs were not required in 41 (51.3%) renovisceral arteries according to preoperative planning. In detail, 15 CAs were left untreated with sufficient distance from the proximal aneurysm extension, 2 additional CAs were already occluded, and 11 SMAs were not involved in the aneurysmal disease. Seven of forty renal arteries were aneurysm-unrelated, resulting in a single renal PGEVAR intervention, four renal arteries were already occluded, and two renal arteries were sacrificed for highly stenotic renal arteries with already preexisting hypoplastic kidneys.

Patients were treated under analgosedation (AS; n = 13) or, in seven patients, under general anesthesia (GA), including two patients with conversion from initial AS to GA. Overall operating times for the initial PGEVAR intervention were 247 ± 129 min (range 135–660), including patients with delayed PG implantation. As expected, operating times for the PGEVAR using more than two PGs or those requiring additional axillary access had longer endovascular procedure times (*p* = 0.013).

Vascular access was obtained by bifemoral percutaneous puncture in combination with supra-aortic access to the left axillary or brachial artery using percutaneous puncture (n = 10) or cut down (n = 3) in 13 patients, and by only bifemoral vascular access without axillary or brachial puncture in 7 patients. Additional procedural data and details about the PGEVAR implantations and parallel graft configurations are shown in Table 2.

### 3.2. Technical Success, Endoleak Rates, and Reinterventions for Endoleaks

Primary technical success was 15/20 (75%). Five patients had postoperatively early T1a (gutter) endoleaks, and three of them had successful reinterventions using coils and liquid glue (Onyx) after 1, 6, and 15 days. The other two T1aELs were considered as low-flow endoleaks with no reinterventions and scheduled for regular surveillance with no late reinterventions or complications. Therefore, the secondary success rate was 18/20 (90%), with no patient having evidence of persistent bleeding or early T1aEL-related complications (Table 3).

The rate of early T1aELs was related to the number of PGs implanted (1 PG vs. ≥2 PGs; *p* = 0.038) and an insufficient oversizing (<30%) of the main aortic stent graft (*p* = 0.038). The proximal main aortic sealing stent graft was oversized between 19 and 38% and the proximal aortic sealing zone length was between 12 and 28 mm. Five patients had insufficient stent graft oversizing and four (80%) of them had an early or late T1aEL. In addition to the described early T1aELs, another T1bEL was observed and treated with a distal PG extension. During the early perioperative period and regular follow-up, no T1cELs or T3ELs were observed. In addition, 10 T2ELs perfused from lumbar arteries and/or patent inferior mesenteric arteries were left untreated without further aneurysm expansion or impact on aneurysm sac behavior (Table 4).

### 3.3. Perioperative and Early Clinical Outcomes

In-hospital mortality until discharge was 1/20 (5%) and perioperative mortality up to 32 days was 3/20 (15%), which was related to perioperative shock, organ failure (n = 1), and predefined therapy limitations for persistent cardiopulmonary and renal dysfunction according to documented patient’s will (n = 2).

Major adverse events (MAE) were documented in eleven patients (55%), including perioperative mortality and stroke in one patient with moderate disability, two myocardial infarctions without need for coronary intervention, and two patients with bowel resection for segmental intestinal ischemia, abdominal compartment syndrome, and non-obstructive mesenteric ischemia, with patent SMA parallel grafts. Five patients had respiratory failure, with prolonged mechanical ventilation and decreased renal function with a need for permanent dialysis in one patient (preoperative GFR already <30 mL/min/1.73 m^2^). Six patients had more than one MAE.

Overall, six early postoperative access complications (6/53 vascular access sites; 11.3%) were observed, requiring endovascular or open surgical reinterventions for access-site hematoma, femoral pseudoaneurysm, brachial nerve and plexus compression, axillary artery dissection, and one patient with a late groin seroma. Two patients had postoperative lower limb ischemia, requiring thrombectomy and lower limb recanalization (Table 3).

### 3.4. Parallel Graft Patency and Reinterventions

Three patients had renal artery parallel graft occlusion after 2, 6, and 44 months; two of them had reinterventions with catheter aspiration and lysis. This was successful in one patient. One occluded renal artery could not be recanalized, and another patient with an occluded renal artery parallel graft had no reintervention because of sufficient perfusion of the kidney via an accessory renal artery. None of these three patients had a relevant decline in kidney function, nor did they require dialysis. Therefore, primary parallel graft patency at 1 and 3 years was 94.8 and 88.2%, while secondary parallel graft patency was 97.4 and 94.1% (Table 4).

### 3.5. Clinical Outcomes During Follow-Up

During follow-up, aneurysm sac behavior, as determined by changes in aneurysm diameter, was analyzed in 19 patients. Three patients showed aneurysm diameter progression, three—aneurysm diameter stability, and thirteen—aneurysm sac regression (68.4%). The overall mean aortic aneurysm diameter determined preoperatively in comparison to the aneurysm diameter at the latest follow-up, analyzed in 19 patients, decreased from 82.7 mm to 68.0 mm; *p* < 0.003; Table 5. As mentioned, three patients had aneurysm progression suspected to be related to a remaining or new onset of a T1aEL in two patients with large aneurysm diameters (120, 90 mm). Of these, one patient was treated with proximal extension of parallel grafts and the other in combination with implantation of a double fenestrated aortic stent graft. The third patient, however, had no detectable T1aELs and declined any reinterventions for worsening health conditions caused by a chronic retroperitoneal infection with proven stent graft infection and died after 3.5 months at the age of 88 years.

Overall mortality was 13/20 (65%), including perioperative mortality (n = 3) and 10 deaths during follow-up. Two of them were considered as late aneurysm-related mortality after 3.5 and 4 months for retroperitoneal (stent graft) infection and postoperative recurrent pneumonia with an aneurysm-related mortality of 25% (5/20, Table 5). The other remaining eight deaths were considered aneurysm-unrelated (two had cancer, two had recurrent respiratory infections with pneumonia, one had a fatal cerebral hemorrhage, and three died from unknown causes of death after stable aneurysm disease at the latest follow-up; these all occurred after the average period of 39 months (range 22–62) following the PGEVAR and at the mean age of 83 years (range: 69–93 y).

### 3.6. Overall Survival and Factors Associated with Long-Term Survival

**Figure 3:**
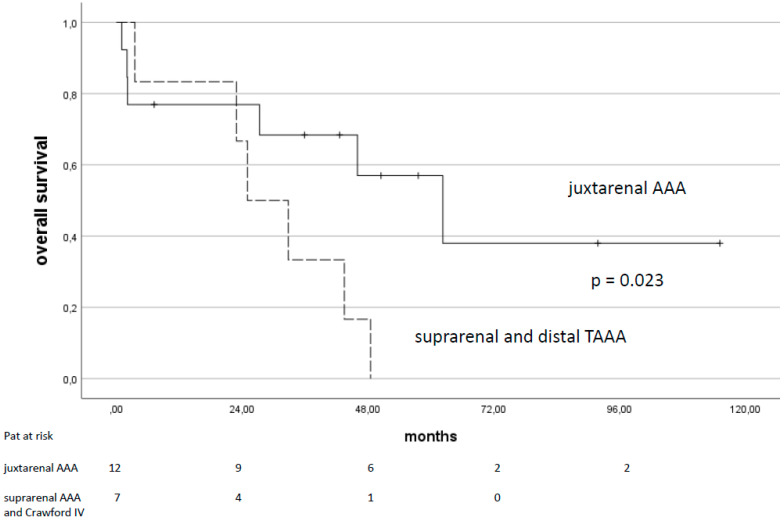
Overall survival of patients with complex abdominal aortic aneurysms treated with PGEVAR comparing juxtarenal AAA and suprarenal/Crawford IV AAA; standard errors were below 0.15 and 0.28, respectively. AAA: abdominal aortic aneurysm.

**Figure 4:**
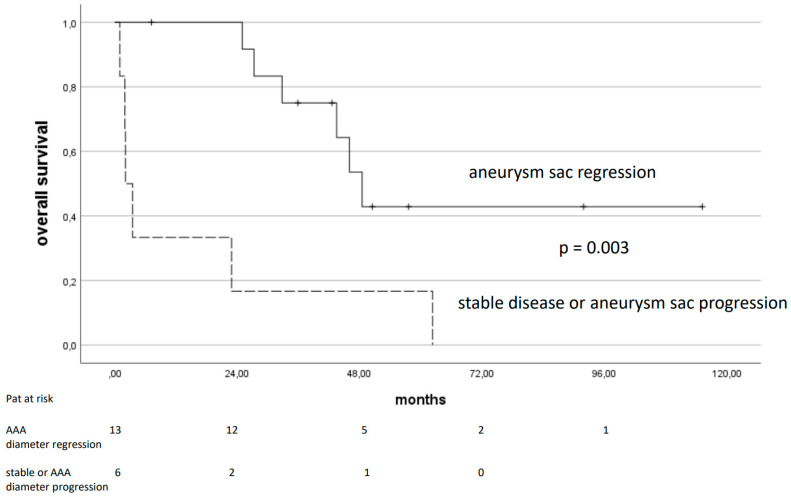
Overall survival of patients with complex AAA treated with PGEVAR in relation to aneurysm sac behavior. Patients’ data after PGEVAR are shown, with aneurysm sac diameter regression displayed in comparison to a combined group of patients with stable aneurysm diameters or aneurysm sac progression; standard errors were below 0.15 and 0.25, respectively. AAA: abdominal aortic aneurysm.

As estimated by the Kaplan–Meier analysis, overall survival was 75% after 1 year, and 53% and 22% after 3 and 5 years. Risk factors significantly associated with a longer overall survival according to a univariable analysis were age (<80 y; *p* = 0.026), juxtarenal aneurysms (*p* = 0.023, Figure 3), absence of major adverse events (*p* = 0.025), and aneurysm sac regression (*p* = 0.003, Figure 4). Other parameters like the initial aneurysm diameter, preoperative hemodynamic instability, preexisting comorbidities, the presence of T1aELs, or the number of parallel grafts implanted (≤2 vs. 3 or 4 PGs) had no impact on long-term outcomes.

## 4. Discussion

Ruptured abdominal aortic aneurysms involving the juxtarenal, suprarenal, or distal thoracoabdominal aortic segments require open surgical or advanced endovascular procedures for immediate aneurysm exclusion with preservation of renovisceral arteries and organ perfusion [6,23,24,25]. Especially for patients with relevant cardiopulmonary comorbidities or with a hostile abdomen after previous major open abdominal surgery, less-invasive endovascular procedures are a valuable therapeutic option for the treatment of these patients who are frequently considered unfit for open surgery [5,26]. Other endovascular therapeutic options like off-the-shelf fenestrated or branched endovascular stent grafts are frequently not available, require dedicated endovascular experience, or might not be suitable according to the individual thoracoabdominal aortic configuration [27,28]. Therefore, the parallel graft EVAR technique with implantation of chimney or periscope parallel grafts for renovisceral organ perfusion can be considered as an important option for endovascular repair of complex ruptured aneurysms without delay [29,30,31,32].

In the present study, we have retrospectively analyzed a cohort of patients with ruptured complex abdominal and distal thoracoabdominal aortic aneurysms, showing that emergent PGEVAR implantations can be performed with a secondary technical success rate of 90% when early T1aELs (gutter endoleaks) are immediately detected and treated by endovascular reinterventions with acceptable perioperative mortality and mid- to long-term outcomes. However, life-long surveillance is mandatory since the secondary expansion of the aortic PGEVAR sealing zone might lead to proximal new-onset endoleak formation or aggravation of preexisting endoleaks, especially in large aneurysms with an increased risk of aneurysm progression and re-rupture [33,34,35,36]. As shown in our study, postoperative endovascular reinterventions for detected T1aELs might result in complete aneurysm sealing, with evidence for aneurysm sac regression being a predictor of aneurysm exclusion with a relevant prognostic impact.

Similar results about the acceptable outcomes of patients urgently treated for complex aortic lesions by renovisceral chimney grafts were reported by Bin Jabr A et al. (2016), including 18 patients with ruptured aneurysms [37]. The authors mention a 10% early T1aEL rate, most of them sealed after endovascular reintervention with a 10% perioperative mortality and a 6% chimney-related mortality with two postoperative ruptures. In their study, the overall outcome was related to a relevant number of sacrificed renal arteries, leading to a high rate of permanent dialysis. Another more recent report from Jernigan EG et al. (2021) has described the outcome of chimney/snorkel endovascular repair for symptomatic and ruptured abdominal aortic ruptures using the Vascular Quality Initiative registry with data from 77 patients, including 35 ruptured aneurysms from 46 states across the USA and Canada [38]. The rate of early postoperative T1aELs was 11.7%, and reinterventions were required in 13% of cases. Major adverse events were observed in 40% of the patients, with a 30-day mortality of 11.4% and a follow-up of 13 months. The authors concluded that long-term data are required to determine the durability of PG endovascular aortic repair for symptomatic and ruptured patients.

Technical success is a critical parameter in publications reporting about the outcome of complex aortic aneurysm repair using parallel grafts, fenestrated, or branched devices. While in some reports, PGEVAR technical success rates were high, with more than 90%, in others, a relevant number of T1aELs are described as requiring reinterventions, and others mention early T1aELs with aneurysm sac perfusion as a technical failure, thus reporting lower technical success rates around 70% [18,39,40]. It is, therefore, important to clearly mention the definition of technical success and give data on primary and secondary technical success rates, considering the T1aELs with or without aneurysm sac perfusion, especially after endovascular repair of ruptured aneurysms.

Within recent years, other advanced endovascular techniques for aortic aneurysm exclusion and preservation of renovisceral perfusion were introduced, and so far, the available mid-term results are promising [41,42]. However, fenestrated stent grafts require several weeks for fabrication. Off-the-shelf, available multi-branched stent-grafts—like the t-branch—are considered to be an established and accepted endovascular outer branch device for elective and emergent aneurysm repair, suitable in about 70% of patients, according to anatomical aneurysm configurations [43]. Nevertheless, this branched device might not properly fit for short paravisceral or juxtarenal aortic aneurysms because of the small aortic diameters and limited space for cannulation and side-branch stent graft implantation. Other options, like physician-modified stent grafts or the in situ fenestration technique using direct needle, radiofrequency, or laser-based puncture techniques for immediate restoration of renovisceral perfusion are not available in every vascular institution and will require dedicated vascular teams with experience and regular training with these techniques [6,44]. Therefore, based on our experience, the described PGEVAR technique seems to still be a valuable option for endovascular repair for a subset of complex ruptured abdominal aortic aneurysms when other therapeutic options are not available or technically and anatomically not feasible.

Long-term data describing the outcomes of patients treated for mainly non-ruptured complex abdominal aortic pathologies within the PERICLES registry were reported by Taneva GT et al. (2021) [45]. The authors show a technical success rate of 88.9%, a chimney graft patency during follow-up of 90.5%, and an overall 5-year survival of 66.1%. Risk factors for late complications were stent graft infection, the absence of an infrarenal aortic neck, or a proximal sealing zone diameter > 30 mm. The reported results of our study, after PGEVAR for ruptured complex abdominal aortic aneurysms, show a secondary technical success rate of 90%, a PG patency rate above 90%, and a lower 5-year survival rate of 22%. The lower overall survival rate after 5 years in our cohort of patients with ruptured complex aortic aneurysms seems to be related to a higher perioperative mortality caused by shock, organ malperfusion, and subsequent relevant postoperative adverse events in ruptured patients with advanced age (≥80 years of age in 45% of our patients).

According to current ESVS guidelines for patients with complex abdominal aortic aneurysms, endovascular repair with fenestrated and branched technologies should be considered as first-line therapy for non-ruptured elective patients. However, in the emergency setting, like in ruptured complex abdominal aortic aneurysms, the parallel stent graft technique is considered in this guideline as a therapeutic option based on patient status, aortic anatomy, local routine, team experience, and patient preference [46]. Regarding long-term durability, data reported in the literature comparing fenestrated EVAR and PGEVAR during elective repair of complex abdominal aortic aneurysms indicate that there is no clear evidence supporting the advantage of one of these two endovascular options over the other [11,28,31,47]. In addition, recent meta-analyses have confirmed the reliability and effectiveness of both endovascular procedures in the elective treatment of complex aortic aneurysms. Furthermore, a recent network meta-analysis indicates no significant difference between the two methods in the short- to medium-term, and the authors stress that many existing studies are often incomplete and of inadequate quality [48]. However, as shown in our study and reported by others, the risk of PGEVAR-related overall mortality is around 10–25%, even in cohorts of ruptured complex abdominal aneurysms with overall acceptable outcome results. Therefore, the PGEVAR technique seems to be an additional valuable endovascular option within the armamentarium of specialized vascular centers, together with other advanced endovascular techniques required for the treatment of complex abdominal or distal thoracoabdominal aortic aneurysms. However, data about the durability and long-term outcomes following PGEVAR implantation for ruptured complex abdominal aortic aneurysms are still limited.

### Limitations

This reported analysis of patients treated with the PGEVAR technique for ruptured juxtarenal, suprarenal, and distal thoracoabdominal aortic aneurysms has several limitations. These include the retrospective nature of this analysis, the small number of patients treated with probably limited evidence for conclusions, and the relevant patient heterogeneity regarding anatomical aortic configuration requiring different endovascular strategies with a possible impact on outcomes. Finally, a selection bias regarding patients considered suitable for this treatment option might have influenced our results without a control group for comparison. However, in the acute emergency situation of ruptured complex abdominal aortic aneurysms, the described PGEVAR technique can be suitable when other treatment options are not available. In addition, based on the focus of our study on juxtarenal, suprarenal, and distal thoracoabdominal aortic aneurysms, the results obtained should not be transferred to more proximally located ruptured Crawford I–III or V aortic aneurysms.

## 5. Conclusions

The PGEVAR technique with implantation of chimney and periscope parallel grafts for treatment of ruptured complex abdominal aortic aneurysm seems to be feasible with an acceptable perioperative mortality and mid- to long-term outcomes. The proximal T1aEL rate of up to 25% is relevant but these ELs can be treated after early detection with immediate reintervention and successful EL sealing in most patients with no persistent bleeding. A sufficient main aortic stent graft oversizing of 30% and an intended proximal PG sealing zone length of 20 mm is recommended in a non-severely angulated visceral aortic segment. Regular surveillance imaging is mandatory following PGEVAR for ruptured complex aortic aneurysms with cautious monitoring for endoleak detection and early reinterventions in large aneurysms and those with aneurysm sac progression. Juxtarenal aortic aneurysms and evidence for aneurysm diameter regression after PGEVAR are relevant predictors for increased long-term survival indicating complete aneurysm exclusion. However, other advanced endovascular options should be additionally considered as treatment options for patients with complex abdominal and thoracoabdominal aortic aneurysms according to the individual anatomical aortic configuration and the endovascular techniques available.

## Figures and Tables

**Table 1:** Demographics, preoperative parameters, and anatomical characteristics.

	Total Patients n = 20
Demographics	
Age (years) mean ± SD	77 ± 9
Sex (male, %)	18 (90)
BMI (kg/m^2^) mean ± SD	31 ± 15
Comorbidities	
Arterial hypertension (%)	17 (85)
Ever smoker (%)	12 (60)
Statin use (%)	6 (30)
Diabetes mellitus (%)	4 (20)
Coronary artery disease (%)	5 (25)
Chronic kidney disease (%)	10 (50)
COPD (%)	1 (5)
Stroke (%)	3 (15)
At Admission	
Blood loss (Hemoglobin < 80 g/dL)	2 (10)
Hypotension (Blood pressure systolic < 80 mmHg)	2 (10)
Acute renal injury (eGFR < 30 mL/min/1.73 m^2^)	2 (10)
INR ≥ 2	3 (15)
Previous operations	
Prior aortic surgery	9 (45)
Thoracic	0
Abdominal	9 (45)
Open aortic repair	3 (15)
EVAR	6 (30)
Indications and anatomical characteristics	
Juxtarenal AAA (%)	12 (60)
Prior endoleak type Ia after EVAR	2
Suprarenal AAA (%)	6 (30)
Crawford IV AAA (%)	2 (10)
Prior abdominal aortic rupture (%)	4 (20)
Preoperative AA diameter (mean ± SD, range)	82 ± 17 mm, 59–120 mm
Coagulation on admission	
APT (%)	3(15)
Dual APT (%)	2 (10)
Anticoagulation (%)	3 (15)
Anticoagulation and APT (%)	2(10)
No anticoagulation or ATP medication (%)	10 (50)

Results are given as numbers (%), mean ± standard deviation (SD), or median with range. BMI: body mass index, COPD: chronic obstructive pulmonary disease, AA: aortic aneurysm, eGFR: estimated glomerular filtration rate, INR: international normalized ratio, APT: antiplatelet therapy, EVAR: endovascular aortic repair.

**Table 2:** Procedural details during PGEVAR.

Total Number	Patients (All) n = 20	Parallel Grafts (All) n = 39
Configuration of parallelgrafts		
Bilateral renal arteries (%)	4 (20)	8
Single Left renal artery (%)	6 (30)	6
Single Right renal artery (%)	1 (5)	1
SMA, bilateral renal arteries (%)	3 (15)	9
SMA, left renal artery (%)	1 (5)	2
SMA, right renal artery (%)	1 (5)	2
Single SMA (%)	1 (5)	1
Celiac artery, SMA (%)	1 (5)	2
Celiac artery, SMA, bilateral renals (%)	2 (10)	8
Aortic Main Body Landing Zone		
Proximal		
Zone 5 (%)	3 (15)	
Zone 6 (%)	6 (30)	
Zone 7 (%)	11 (55)	
Distal		
Zone 9 (%)	4 (20)	
Zone 10 (%)	16 (80)	
Time point of PG implantation for rEVAR		
Chimney/Periscope during initial EVAR (%)	17 (85)	
Chimney/Periscope after 1–10 days (%)	3 (15)	
for Type Ia Endoleak (%)	2 (10)	
for short infrarenal neck (%)	1 (5)	
Number of parallel grafts	39 (23 Chimneys, 16 Periscopes)	
Patients with 1 (%)	8 (40)	
2 (%)	7 (35)	
3 (%)	3 (15)	
4 (%)	2 (10)	
Additional details		
Access Vessel		Access sites
Axillary and femoral (%)	13 (65)	39
bifemoral (%)	7 (35)	14
Femoral percutaneous access/cut down	12/8	
Axillary percutaneous access/cut down	10/3	
Length of procedure (minutes), median (range)	170 (60–660)	
General Anesthesia (%)	9 (45)	
Analgosedation (%)	11 (55)	

Results are given as numbers (%), mean ± standard deviation (SD), or median with range. SMA: superior mesenteric artery.

**Table 3:** Perioperative outcomes.

	Total n = 20
Technical Success	
Primary technical success (%)	15 (75)
Secondary technical success (%)	18 (90)
In-hospital outcomes	
Major adverse events	11 patients (55)
Acute renal failure (%), temporary/permanent dialysis	8 (40%), 3/1
Myocardial infarction (%)	2 (10)
Mesenteric ischemia (%)	2 (10)
Spinal cord ischemia temporary/permanent (%)	0
Multi-organ failure (%)	1 (5)
Access complications (%)	6 (30)
Secondary procedures for	
persistent aortic hemorrhage	0
bowel resection	2
peripheral embolic complications	1
access-site complications	6 (53 access sites; 11.3%)
Hospital and ICU stay	
Length of hospital stay (d) median (range)	17 (8–76)
Length of ICU stay (d)	3.5 (1–53)
Perioperative mortality	
in-hospital mortality (%)	1 (5)
perioperative (32 d) mortality (%)	3 (15)

**Table 4:** Endoleaks, endoleak-related reinterventions, and parallel graft patency.

Endoleak (EL)	n (%)	Reinterventions	Success
Type Ia EL	7 (35)	5/7 *	5/5 (100%)
Early (<30 days)	5 (25)	3/5 *	3/3 (100%)
Late (≥30 days)	2 (10)	2/2	2/2 (100%)
Type Ib Endoleak	1 (5)	1/1	1/1 (100%)
Type II Endoleak	10 (50)	-	-
Type III Endoleak	0	-	-
Reinterventions for PG instability			
Overall	3/39 PGs (7.7%)		
Successful reinterventions	1/3 (33%)		
	PG target vessel	Time to RI	new dialysis
Patient [no. 2]	LRA	2 months	no
Patient [no. 11]	RRA	44 months	no
Patient [no. 16]	RRA	5.5 months	no
Secondary PG instability of target vessel			
Celiac artery	0/3 (0%)		
SMA	0/9 (0%)		
RRA	1/11 (9.1%)		
LRA	1/16 (6.3%)		
Parallel graft patency			
Primary patency rate (1 year)	37/39 (94.8%)		
Secondary Patency rate (1 year)	38/39 (97.4%)		
Primary patency rate (3 years)	15/17 (88.2%)		
Secondary patency rate (3 years)	16/17 (94.1%)		

Results are given as numbers (%), mean ± standard deviation (SD), or median with range, * one patient with low-flow EL without aneurysm progression and one patient with low-flow EL and suspected stent graft infection. SMA: superior mesenteric artery, LRA: left renal artery, RRA: right renal artery, PG: parallel grafts.

**Table 5:** Mid- to long-term outcomes.

Follow-up (months), median (range)	34 (0–115)
Aneurysm sac behavior (n = 19) mean ± SD	−10 mm ± 17 mm
Diameter regression < −5 mm	13 (68.4), (−14 mm ± 14 mm)
Stable aneurysm diameter (−5 mm to +5 mm)	3 (15.8)
Diameter increase > 5 mm (%),	3 (15.8), (+9.7 mm ± 0.5 mm)
Preoperative AA diameter (mean ± SD, range)	82 mm ± 18 mm, 59–120 mm
Postoperative AA diameter (mean ± SD, range)	68 mm ± 26 mm, 37–125 mm
	(*p* = 0.003)
Mortality and survival	
Overall mortality (%)	13 (65)
Aneurysm-related mortality (%)	5 (25)
1-year survival rate (%)	14 (70)
3-year survival rate (%)	7 (35)
5-year survival rate (%)	3 (22)

Results are given as numbers (%), mean ± standard deviation (SD), or median with range; survival estimates are based on Kaplan–Meier analysis.

## Data Availability

The data presented in this study are available on request from the corresponding author. The data are not publicly available due to regional regulations.
